# Natural Vocalizations in the Mammalian Inferior Colliculus are Broadly Encoded by a Small Number of Independent Multi-Units

**DOI:** 10.3389/fncir.2015.00091

**Published:** 2016-02-01

**Authors:** Dominika Lyzwa, J. Michael Herrmann, Florentin Wörgötter

**Affiliations:** ^1^Max Planck Institute for Dynamics and Self-OrganizationGöttingen, Germany; ^2^Institute for Nonlinear Dynamics, Physics Department, Georg-August-UniversityGöttingen, Germany; ^3^Bernstein Focus NeurotechnologyGöttingen, Germany; ^4^Institute of Perception, Action and Behavior, School of Informatics, University of EdinburghEdinburgh, UK; ^5^Institute for Physics - Biophysics, Georg-August-UniversityGöttingen, Germany

**Keywords:** inferior colliculus, vocalizations, neural discrimination, neural correlations, multi-unit cluster, guinea pig, efficient encoding

## Abstract

How complex natural sounds are represented by the main converging center of the auditory midbrain, the central inferior colliculus, is an open question. We applied neural discrimination to determine the variation of detailed encoding of individual vocalizations across the best frequency gradient of the central inferior colliculus. The analysis was based on collective responses from several neurons. These multi-unit spike trains were recorded from guinea pigs exposed to a spectrotemporally rich set of eleven species-specific vocalizations. Spike trains of disparate units from the same recording were combined in order to investigate whether groups of multi-unit clusters represent the whole set of vocalizations more reliably than only one unit, and whether temporal response correlations between them facilitate an unambiguous neural representation of the vocalizations. We found a spatial distribution of the capability to accurately encode groups of vocalizations across the best frequency gradient. Different vocalizations are optimally discriminated at different locations of the best frequency gradient. Furthermore, groups of a few multi-unit clusters yield improved discrimination over only one multi-unit cluster between all tested vocalizations. However, temporal response correlations between units do not yield better discrimination. Our study is based on a large set of units of simultaneously recorded responses from several guinea pigs and electrode insertion positions. Our findings suggest a broadly distributed code for behaviorally relevant vocalizations in the mammalian inferior colliculus. Responses from a few non-interacting units are sufficient to faithfully represent the whole set of studied vocalizations with diverse spectrotemporal properties.

## 1. Introduction

Vocalizations are spectrotemporally varying sounds which display a wide spectrum of acoustic properties, such as amplitude and frequency modulations, harmonics and temporal correlations. These natural sounds are well suited for studying the auditory system, since it was suggested that neurons are adapted to process them (Rieke et al., 1995). Thus, they might trigger responses which are not elicited by artificial or simple acoustic stimuli. Guinea pigs are very communicative and display a rich repertoire of behaviorally relevant vocalizations (Berryman, 1976). We address the question how the inferior colliculus (IC) of these mammals encodes species-specific vocalizations. The inferior colliculus is the main processing station in the auditory midbrain (Irvine, 1992) and integrates projections from essentially all ascending auditory brainstem nuclei (Aitkin and Phillips, 1984; Malmierca et al., 2002). Apart from being a center of convergence, further sound feature extraction is presumably performed in the IC (Joris et al., 2004). The central nucleus of the inferior colliculus (ICC) is essential for extracting time-varying spectrotemporal information (Escabí and Schreiner, 2002) and therefore might be important for processing complex sounds such as speech and vocalizations.

Our study is based on multi-unit activity which is the collective response mainly from neighboring neurons that span one order of magnitude. Investigating the encoding of natural sounds on the level of multi-unit clusters might have the advantage that this response is an integrated activity which could reflect local system processing of the ICC. Furthermore, multi-unit clusters respond stronger to natural sound than single neurons (Grace et al., 2003) and natural sound stimuli can be more accurately discriminated based on these responses than based on single neuron responses (Engineer et al., 2008). We investigate the encoding of natural stimuli using neural discrimination and address three questions.

(1) The average value of the neural discrimination across a whole set of vocalizations has been tested previously for grasshopper auditory periphery receptor single cells (Machens et al., 2003), and between amplitude modulation frequencies for receptor single cells, local and ascending interneurons (Wohlgemuth and Ronacher, 2007). Furthermore, it has been tested between songs for single neuron responses of zebra finches, in the homologous structure to the inferior colliculus in mammals (Schneider and Woolley, 2010). Schneider and Woolley have shown that single neuron responses could be used to discriminate between 11 bird songs with up to perfect performances. The total discrimination averaged across the whole set of communication calls did not depend on the frequency tuning, i.e., the best frequency of the neuron (Schneider and Woolley, 2010). However, preferred encoding of individual calls in specific frequency regions might exist, but was not detectable in the averaged discrimination. Thus, the encoding needs to be analyzed for individual vocalizations. In the ICC, response heterogeneity has been shown within (Schreiner and Langner, 1988; Holmstrom et al., 2010; Baumann et al., 2011) and across (Rose et al., 1963; Merzenich and Reid, 1974; Suta et al., 2003) isofrequency laminae. An isofrequency lamina contains neurons with similar best frequencies (Schreiner and Langner, 1997; Malmierca et al., 2008). In this work, the optimal encoding of individual vocalizations with their specific spectrotemporal content is compared across the best frequency gradient. A purely linear mapping of the vocalizations' spectral contents along the best frequency gradient is unlikely due to nonlinear processing mechanisms (McAlpine, 2004; Escabí et al., 2005; Calabrese et al., 2011).

Individual vocalizations in the guinea pig ICC have been shown to be encoded based on their spectrotemporal patterns (Suta et al., 2003). For four vocalizations and within four frequency intervals, Suta et al. (2003) demonstrated a dependence of the spike-rate on the neuron's characteristic frequency. However, it has also been shown that spike-timing information is crucial for neural discrimination of vocalizations and intelligibility of speech (Shannon et al., 1995; Schnupp et al., 2006). To compare optimal encoding of individual vocalizations with the neuron's preferred frequency, we use spike train trials and a fine frequency resolution of 36 intervals across the whole set of 11 vocalizations.

Holmstrom et al. (2010) discriminated single neuron responses to natural vocalizations and their modified versions in the mouse ICC. They showed that neurons display heterogeneous responses to each perturbation of acoustic features in these stimuli, and different neurons responded differently to the same vocalization. They also showed that heterogeneous neural responses in the mouse inferior colliculus efficiently encode vocalizations. However, the specific encoding remains an open question, because either heterogeneously distributed neural responses (Holmstrom et al., 2010) could lead to individual vocalizations being encoded equally well across the ICC, or the vocalizations might be encoded more topographically (Suta et al., 2003), following the organization of spectrotemporal preferences in the ICC (Rose et al., 1963; Merzenich and Reid, 1974; Schreiner and Langner, 1988). We tested this alternative by comparing encoding of individual vocalizations along the frequency gradient of the ICC.

(2) The ability of single neurons to encode vocalizations in the auditory midbrain has been shown to vary (Schneider and Woolley, 2010). Previous studies have investigated the encoding of combined responses (Engineer et al., 2008; Schneider and Woolley, 2010). In the zebra finch, Schneider and Woolley (2010) have found optimal separability of responses for combining spike trains from 2 to 5 single neurons with similar tuning, and suggested that pooling reduces trial-to-trial variability and therefore increases separability. Engineer et al. (2008) have shown that the ability of a group of neurons to discriminate outperforms the discrimination ability of a single neuron for the primary auditory cortex of rats. The rats were presented with human speech sounds, which could result in different degrees of encoding accuracy than for species-specific vocalizations.

In general, single neurons do not provide enough discriminative information to perfectly distinguish vocalizations. However, a large population of neurons responding simultaneously in order to encode vocalizations would not agree with the efficient encoding hypothesis (Barlow, 1961). This leads us to ask how distributed the encoded information is for the vocalizations in the mammalian ICC. To address this question we investigate whether discrimination accuracy changes when combining responses from an increasing number of multi-unit clusters, and if the accuracy is effected by whether the multi-unit clusters have similar or dissimilar frequency tuning. Additionally, we investigate encoding for combining spectral, temporal or the joint spectral and temporal information from different multi-units clusters.

(3) Third, we examined whether temporal response correlations, i.e., correlated trial-to-trial-variability, effect neural discrimination. The neural activity of units involved in the representation of the vocalization could be coupled, yielding temporal response correlations which are also referred to as noise correlations (Averbeck et al., 2006) or neural correlations (Eggermont, 1994). These correlations could lead to more efficient encoding. Whether this correlated activity, which is due to interactions between the neurons, increases or decreases encoding efficiency is not clear (Averbeck et al., 2006). Both, response correlations and neural correlations have been shown to be destructive or invariant (Nirenberg et al., 2001), or favorable (Abbott and Dayan, 1999; Pillow et al., 2008; Ecker et al., 2011; Da Silveira and Berry II, 2013) to encoding of sensory stimuli. The effect of neural correlations might depend on the specific neural system and its correlational structure (Averbeck et al., 2006). Neural representations also have been proposed to decrease in redundancy from peripheral to cortical structures (Barlow, 1972). It has been shown for the primary auditory cortex of the zebra finch that temporal response correlations for spike trains longer than 250 ms do not change encoding (Wang et al., 2007). However, this has not been investigated previously for short-time spike train durations for vocalizations in the mammalian ICC (thus behaviorally crucial durations), although one could expect correlations across neurons on short-time scales in the ICC (Chen et al., 2012a). We tested the hypothesis that temporal correlations of simultaneously recorded neurons facilitate neural discrimination.

In this study we investigated encoding accuracy of individual vocalizations across the tonotopy, whether groups of multi-unit clusters encode vocalizations better than one multi-unit cluster, and whether temporal correlations facilitate encoding.

## 2. Materials and methods

### 2.1. Electrophysiology

Neural recordings from the central nucleus of the inferior colliculus (ICC) of 11 adult male and female Dunkin Hartley guinea pigs were taken while acoustically presenting conspecific vocalizations to the left ear. The experimental setup, including sound calibration, is described in detail elsewhere (Rode et al., 2013). For the recording, double-shank arrays (shank distance was 500 μm with 16 contacts linearly spaced at 100 μm, on each shank) and 4-double-tetrode arrays (shank distance of 500 μm, channel distance within tetrode of 25/82 μm) were used (impedances: 0.5–1 MΩ at 1 kHz; NeuroNexus Technologies, Ann Arbor, MI). With these arrays the neural activity from the contralateral ICC was recorded simultaneously from 32 different sites (channels). The multi-site electrode array was introduced under an angle of 45° dorsolateral along the gradient of best frequencies, the tonotopic gradient. Frequency response areas were computed in response to pure tone stimulation. The tone-evoked best frequency (BF), the stimulus frequency eliciting the highest spike-rate at each given intensity, ranged from 0.5 to 45 kHz. The same frequency range was covered by linear-double shank and double-tetrode recordings. The guinea pigs were anesthetized with Ketamine and stereotactically fixed with ear tubes through which the sound was presented directly to the eardrum. A total of 11 different vocalizations were played with intensities of 30–70 dB SPL in steps of 10 dB SPL. Unless stated otherwise, the recordings analyzed are the ones at 70 dB SPL stimulus intensity, as they show the strongest response. For each vocalization 20 trials were recorded at a given intensity. Recordings of 1 s and 1.6 s duration were taken with a TDT Tucker Davis System with a sampling rate of 24.414 kHz. For each animal (*N*_animals_ = 11), the multi-site electrode array was inserted into 3 or 4 different positions for recording.

### 2.2. Stimuli

Vocalizations were recorded, with a sampling rate of 97.656 kHz, from male and female Dunkin Hartley guinea pigs (details can be found in Rode et al., 2013).

**Figure 1:**
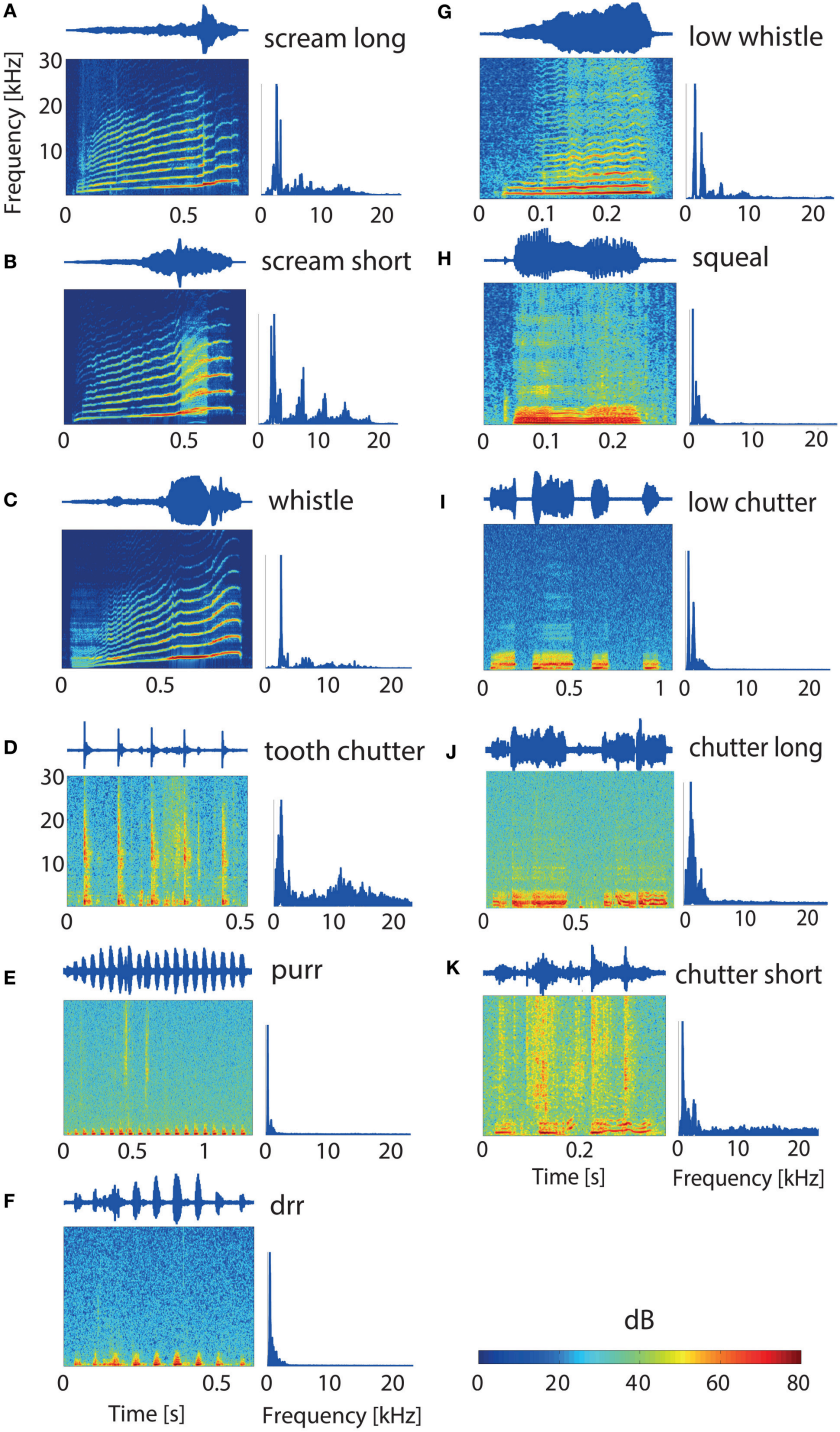
**Guinea pig vocalizations**. Waveforms, spectrograms, and power spectrums for each of the 11 studied vocalizations **(A–K)**. Vocalization durations vary from 0.3 to 1.3 s **(H,E)**. The “tooth chatter,” “purr,” and “drr” **(D–F)** have envelope periodicities of 10.3, 15.4, and 14 Hz, respectively. The latter ones have only low frequency contents below 3 kHz. Both “screams” and the “whistle” **(A–C)** show very distinct harmonics and broad frequency distributions. The vocalizations display quite different spectrotemporal properties.

The 11 vocalizations studied here constitute a representative set of guinea pig communication calls and give information about the animal's behavioral state (Berryman, 1976). The waveform, spectrogram and power spectrum of the vocalizations used in this study are shown in Figure 1. These spectrotemporally varying complex sounds display a large variety of frequency modulations, frequency ranges, and envelope types. The spectrograms of some of the vocalizations display harmonics (Figures 1A–C,G,H). The waveform has a simple periodic shape for some vocalizations (“tooth chatter,” “purr,” “drr,”Figures 1D–F) and is quite complex for others (e.g., “squeal,” “low whistle,” Figures 1H,G). Some vocalizations have a frequency range of up to 30 kHz. The impulse-response function of the system (loudspeaker-tube-mold of ear canal) was measured. Then, the vocalizations were filtered with the estimated inverse transfer function in order to compensate for the effects of playing the stimuli through the loudspeaker-tube-ear-mold system and presented to the guinea pigs at a sampling rate of 195.31 kHz (Rode et al., 2013). The vocalizations were played 20 ms after recording onset and vary in duration between 300 and 1300 ms.

For the analysis, the main spectral content of the vocalization was obtained by computing the power spectrum and integrating the power for frequency bands, *f*_*N*_, which are centered around the recorded multi-units' best frequencies, *f*_*N*_ = {0 − 0.25}, {0.25 − 0.55}, …, {38 − 45.5} kHz. The power for each vocalization was normalized and its logarithm displayed. Pure tone stimulation was used to identify frequency response areas. A total of 40 stimulus frequencies, ranging between 0.5 and 45 kHz, with a ramp rise and fall time of 5 ms each and a duration of 50 ms, were presented 20 ms after recording onset.

### 2.3. Preprocessing of recordings

The impedance of the electrodes allows us to capture the compound response of several neighboring single neurons recorded from one site, which is referred to as a multi-unit or multi-unit cluster. We used the offline spike-sorting program *WaveClus* (Quian Quiroga et al., 2004) to sort and separate spikes according to spike waveform. This spike-sorting program carries out a wavelet-analysis on the recordings, and based on the wavelet coefficients, action potentials are clustered.

We performed spike-sorting on a subset of the recorded multi-unit cluster responses which covers the analyzed best frequency range. All 20 trials were concatenated, filtered between 300–3000 Hz and spikes were assigned where voltages exceeded a threshold of three standard deviations of the ongoing activity. From each multi-unit within the analyzed set, 3–5 clusters of sorted responses with sizes ranging from 100 to 20,000 spikes were obtained. In some cases, a group of spikes of the same magnitude could not be attributed to any of these sorted clusters. Within the found sorted clusters, 50–5000 spikes displayed inter-spike-intervals of less than 3 ms, which indicates that separation into single units was not possible. Thus, the responses investigated and interpreted in this paper are from neural groups comprising at least 3–5 single neurons and smaller contributions from neurons that are farther away from the recording electrode and are not distinguishable. Note that it is possible that different sub-groups of multi-unit clusters respond to different vocalizations.

Spiking multi-unit activity was obtained from the recorded voltage traces by applying a Butterworth filter with a passband of 300–3000 Hz and thresholding 3 (*z* = 3) standard deviations above the ongoing activity. From the spontaneous activity, the mean (μ) and standard deviation (σ) are computed. Activity exceeding the threshold Θ, which is a linear function of the standard deviation (Θ = μ + *zσ*), is counted as a spike. No refraction time between spikes was assumed, as these likely originate from different single units. This spontaneous or ongoing activity was acquired from the first 20 ms of each recording, during which no stimulus was presented, in order to account for different spontaneous rates of the multi-unit clusters and adaptation effects over time. Multi-unit spike trains were binned at 1 ms and convolved with a filter function, *f*(*t*) = *t* · exp(α · t), with time *t*, to mimic the time course of excitatory postsynaptic potentials (EPSP) (van Rossum, 2001), as used by Machens et al. (2003). The full width at half maximum α, of the EPSP-like function was chosen to be 3 ms. This is the smallest time scale, thus the highest temporal resolution of the window found by Machens et al. (2003) to yield maximum discrimination performances between spike trains in response to vocalizations in the grasshopper auditory system. To obtain local field potentials, the voltage traces were Butterworth filtered in a range between 0.5 and 500 Hz (Pettersen et al., 2012).

### 2.4. Neural discrimination

**Figure 2:**
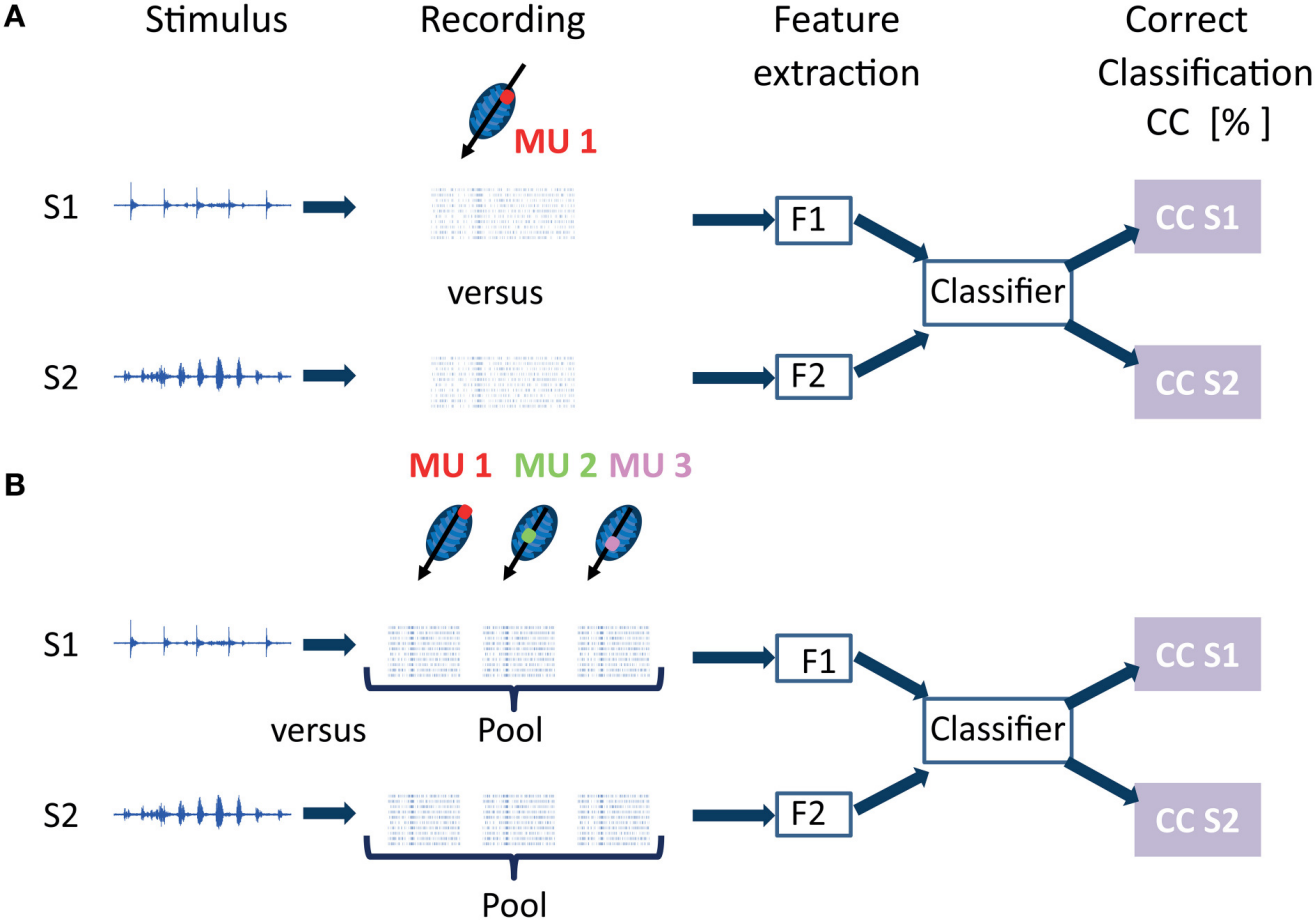
**Classification procedure for neural discrimination between responses elicited by different vocalization stimuli**. In this schematic, the case for discrimination between 2 vocalizations out of 11 is displayed. **(A)** Recordings from one multi-unit cluster (MU 1) to stimuli S1 and S2 are preprocessed, to yield either spiking activity or local field potentials. Features (F1 and F2) are extracted for *n*_trial_ response trials (*n*_trial_ = 20) to stimulus S1 or S2. The features are passed to a classifier which yields the percentage of correctly assigned responses for stimuli 1 and 2, the percentage correct classification (*CC*). This classification procedure is performed independently for each multi-unit cluster with its specific frequency tuning. **(B)** Responses from several multi-unit clusters are pooled and discriminated. In the schematic, responses from three multi-unit clusters (MU1, MU2, MU3) which are along the tonotopic gradient and have different best frequencies (BF), are pooled, either by adding up trials from different multi-unit clusters or by concatenating them. From these combined response trials to stimuli S1 and S2, features (F1 and F2) are extracted and classified to obtain percentage correct assignment of the pooled responses.

Neural discrimination is the ability to distinguish different stimuli based on the neural responses they elicit. These responses are classified according to a chosen distance metric. In the following, when referring to “classification” it is meant to imply “neural discrimination.” The percentage of correctly classified responses is used to quantify how accurately the neural data allows one to discriminate between the different vocalizations. The procedure to test for separability between responses to different stimuli from one multi-unit cluster is schematized in Figure 2A for two vocalization stimuli, and consists of data preprocessing, feature extraction and classification. Separability of responses was tested independently for each multi-unit cluster from linear double-shank recordings and from double-tetrode recordings. Whereas multi-unit responses from the former are recorded along the best frequency gradient, and cover a broad range of best frequencies, the responses from the latter are from a few neighboring isofrequency laminae, and several multi-unit clusters have similar frequency tuning. This allowed us to investigate whether the ability to discriminate between individual vocalizations (or between groups of vocalizations) varies with multi-unit frequency tuning.

To separate responses to different stimuli, features obtained from the time courses were employed. The tested features were: mean response rates (for LFP) and firing rate (for spiking activity) across 300 ms, a 6-dimensional feature vector, containing respective mean rates over six consecutive periods of 50 ms, yielding a finer resolution of the response rate over the previous mean rate, and, finally, the five most prominent frequencies of LFP-recordings obtained from the power spectrum. The first two metrics have been applied previously for successful discrimination of responses to vocalizations (Machens et al., 2003; Portfors et al., 2009). Features were obtained for each of the *n*_trial_ = 20 trials, which were divided into test and training sets and entered to a classifier. A 10-fold cross-validation with 10% test data was performed for each classification, and yielded an estimate of the error. Tested classifiers included linear discriminant analysis (LDA, which fits a normal density to each class and estimates the combined covariance matrix), a naive Bayesian classifier (which estimates a diagonal covariance matrix) and a nearest neighbor classifier, using Euclidean distance (Duda et al., 2000). As a further approach we used the correlation of spiking responses to be classified (test trials) with the labeled spiking responses (training trials). Correlation values serve as features and the test trial was assigned to the class which yielded the highest correlation value. A correlation-based similarity measure of spike trains (Schreiber et al., 2003) has been employed earlier for neural discrimination of single neurons and groups of neurons (Wang et al., 2007).

Temporal information has been shown to be crucial for the comprehension of speech (Shannon et al., 1995) which is spectrotemporally varying complex sound as are vocalizations. Here, we computed the degree of correlation between two spike trains from one multi-unit cluster as an index of the responses' temporal information. Correlation values were computed as the coefficient of correlation of two EPSP-spike trains *x*(*t*), *y*(*t*), of length *n*.

(1) $$\begin{array}{l} Corr=\frac{\sum_{t=1}^{n}\left(x\left(t\right)-\langle x\rangle \right)\cdot \left(y\left(t\right)-\langle y\rangle \right)}{\sqrt{\sum_{t=1}^{n}{\left(x\left(t\right)-\langle x\rangle \right)}^{2}\sum_{t=1}^{n}{\left(y\left(t\right)-\langle y\rangle \right)}^{2}}}. \end{array}$$

The correlation values were computed at zero lag, because even though response latencies do vary across multi-unit clusters with different spectrotemporal preferences (Langner et al., 1987), here, responses from the same multi-unit cluster were compared. The correlation approach allows us to compare temporal responses at a resolution of 3 ms.

**Figure 3:**
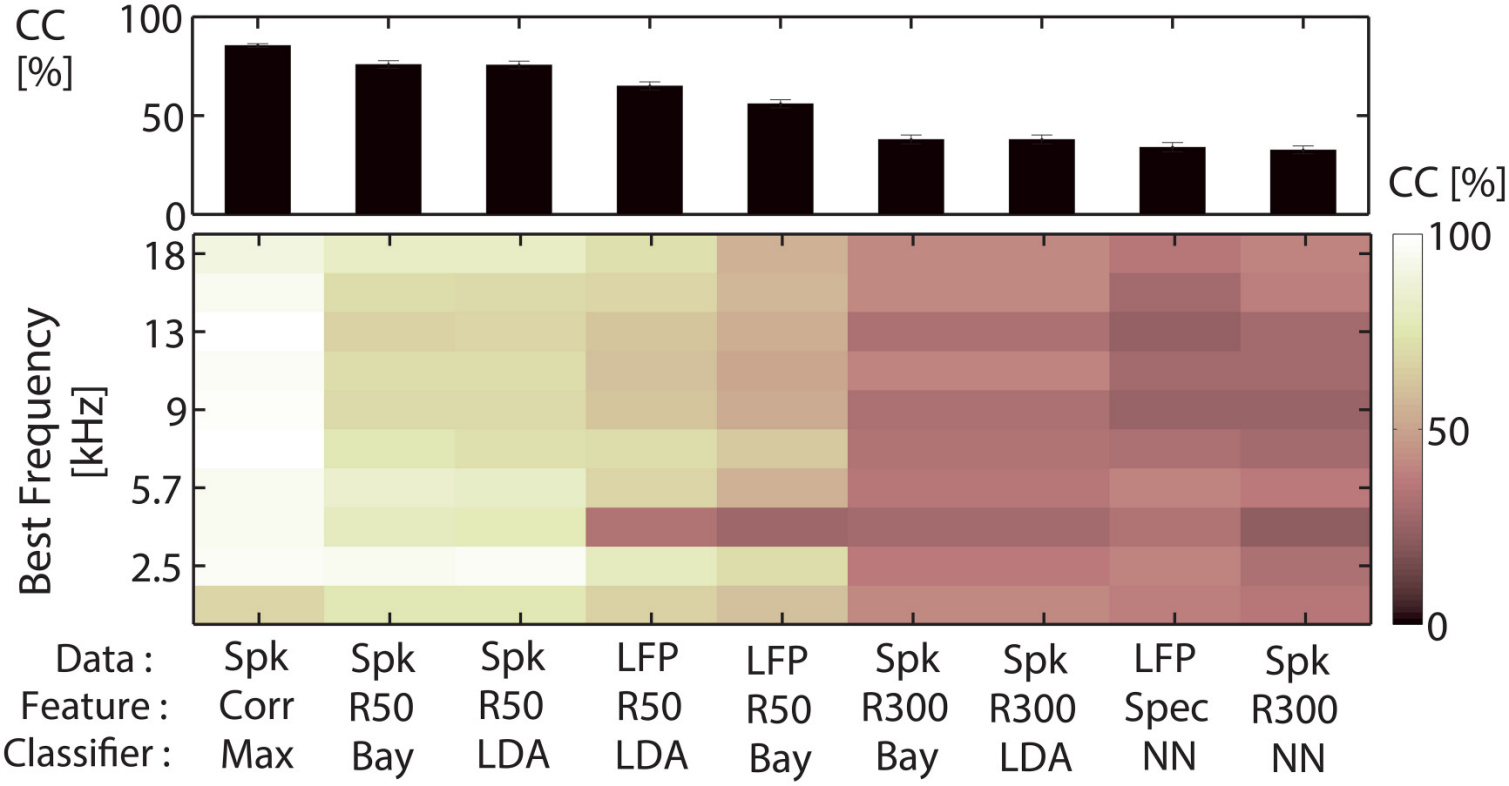
**Comparison of classification performances for different (data type, feature, classifier)-combinations**. Comparison of classification performances for nine (data type, feature, classifier)-combinations, for a classification between all 11 vocalizations on 300 ms of recording. Data types are EPSP-spike trains (Spk), and local field potentials (LFP). Features are correlation values (Corr), the whole segment of spike trains or LFPs, the average (firing) rate of 300 ms (R300) or six average (firing) rates of each 50 ms (R50), or the five most important response frequencies (Spec). Classification was performed by choosing the maximum value (Max), a naive Bayes classifier (Bay), linear discriminant analysis (LDA) or nearest neighbor classification using Euclidean distance (NN). **Top**: Correct classification averaged across 10 multi-unit clusters from one recording are displayed for each of the parameter combinations. Correlation of spike trains outperforms the other classification procedures. **Bottom**: Classification performance for the individual 10 multi-unit clusters with different best frequencies, spanning a frequency range of 0.5–18 kHz. Classification does not vary systematically with the multi-unit cluster's best frequency.

**Figure 4:**
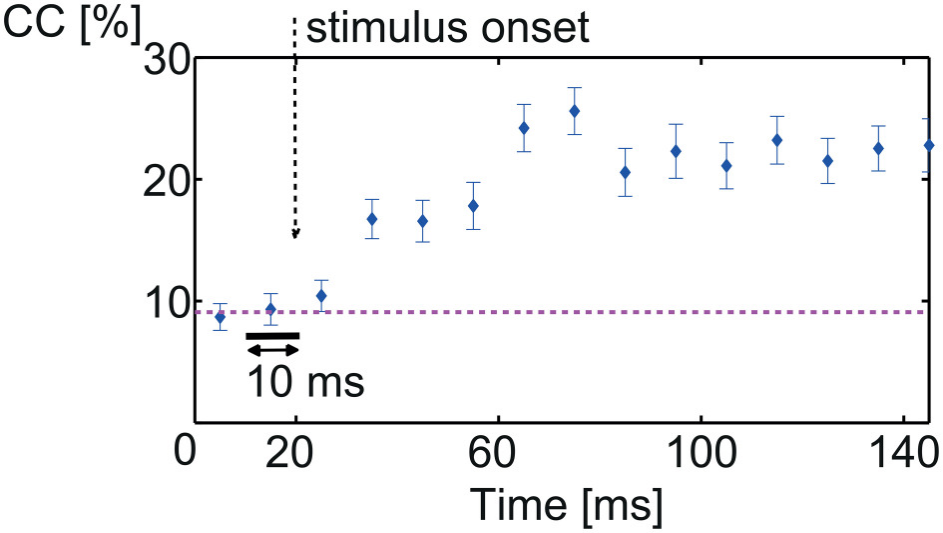
**Onset of discrimination**. Percentage correct classification between all 11 vocalizations of consecutive spike trains of 10 ms. Low correct classification values are due to the relatively short spike train length. Before stimulus onset, classification was at chance level (9.1%, pink dashed line). After stimulus onset, classification performance gradually increased to maximum 25% correct classification. Discriminative information is present only after stimulus onset.

**Figure 5:**
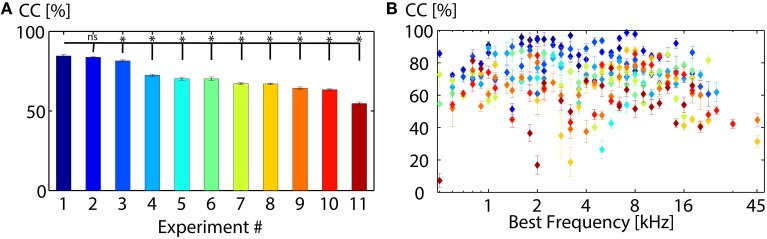
**Classification between 11 vocalizations varies across animals. (A)** Average correct classification values of all recordings from one experiment (animal). Classification performances vary significantly, but are similar for some experiments (Experiment 1 and 2). **(B)** Average correct classification value of each experiment for each best frequency. Higher classification values do not correlate with best frequencies but vary for different animals (^*^ denotes significant, ns denotes non-significant results; mean, Wilcoxon-Mann-Whitney test *p* = 0.05).

**Figure 6:**
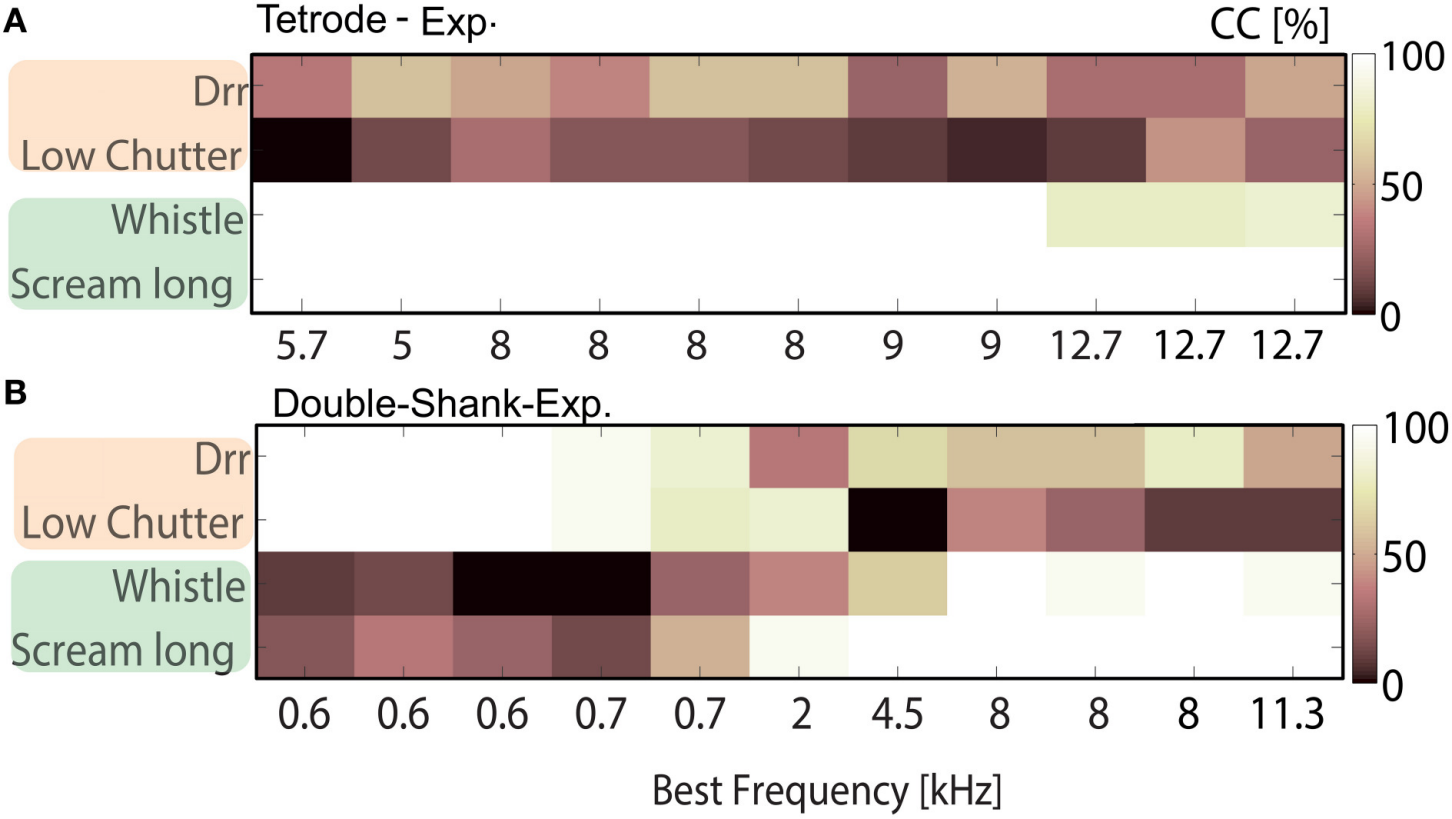
**Tradeoff of best neural discrimination with best frequency**. Classification between responses to low (“drr,” “low chutter”) and middle to high (“whistle,” “scream long”) frequency containing vocalizations for multi-unit clusters from one shank **(A)** from a double-tetrode array recording with several units of similar frequency tuning, BF = 5.7–9 kHz; and **(B)** from a recording along the best frequency gradient with a broad range of best frequencies, BF = 0.6–11.3 kHz. The “drr” and “low chutter” are nearly perfectly encoded for BFs up to 0.7 kHz, whereas the “whistle” and “scream long” are very poorly discriminated in this range. For BFs above 4.5 kHz, the “whistle” and “scream long” are perfectly discriminated but “drr” and “low chutter” are frequently confused. A clear transition from nearly perfect discrimination of low to middle-high frequency vocalizations is present between 0.7 and 4.5 kHz.

**Figure 7:**
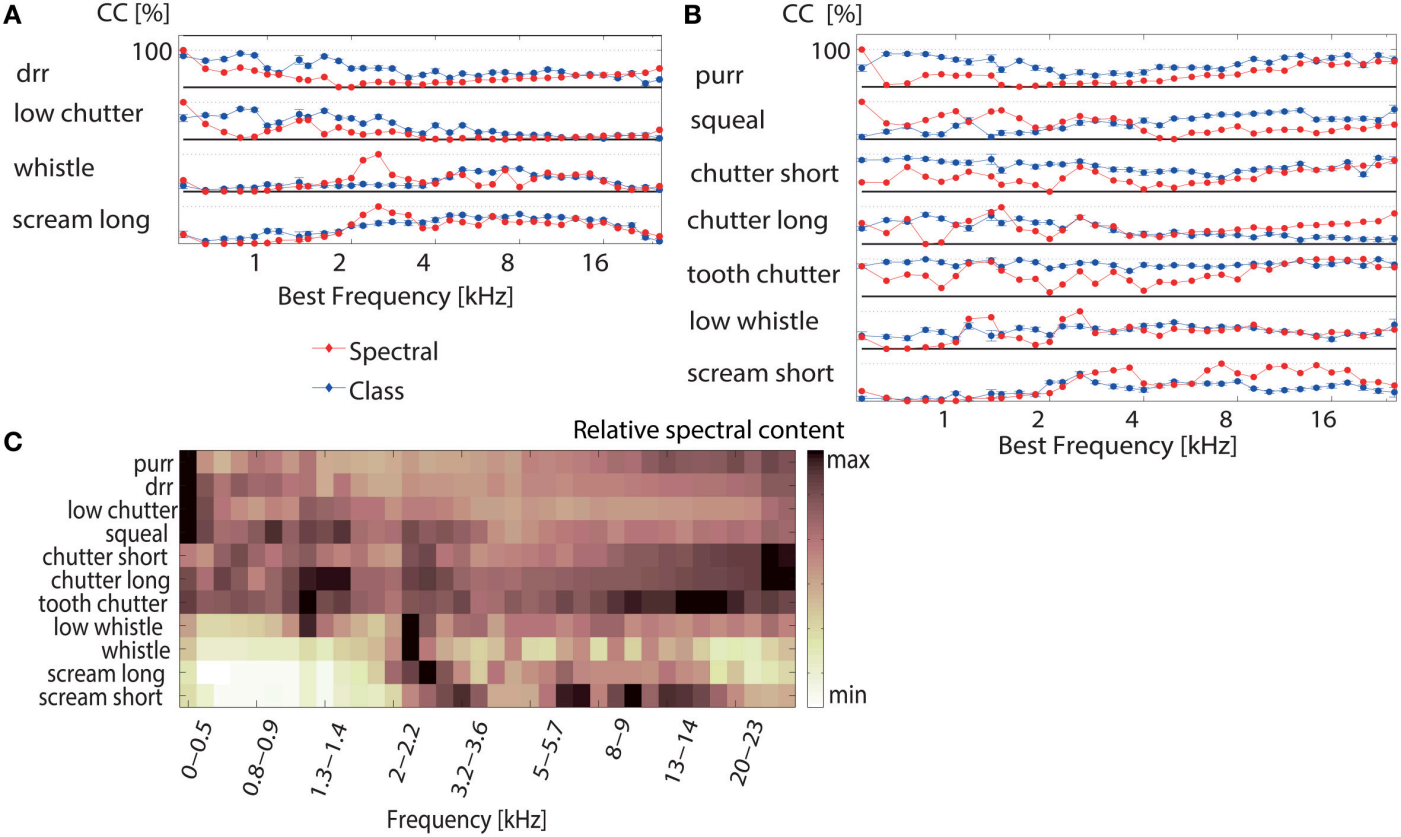
**Correct classification for individual vocalizations depends on the multi-unit cluster's best frequency. (A)** The correct classification (Class _
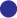
_) of individual vocalizations for discrimination between all 11 vocalizations (averaged across 1152 multi-unit clusters) is displayed for the “whistle,” “scream long,” “drr,” and “low chutter.” The black line denotes 100% correct classification for each vocalization. Correct classification is high for the “whistle” and “scream long” for multi-unit clusters with middle to high BFs (5–15 kHz, 2–20 kHz) and correct classification is high for the “drr” and “low chutter” for multi-unit clusters with low BFs (<4 kHz). The high correct classification values are distributed across the best frequency range. The relative frequency content for each vocalization is shown (Spectral _
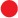
_). The match between relative frequency content and correct classification is perfect at some frequencies. (B) Correct classification values as in (A) are displayed for the remaining vocalizations. A distribution of high correct classification values across best frequencies also exists. The “long chutter” is correctly discriminated by low BF-units and the “low whistle,” “squeal” and “short scream” are correctly discriminated by responses from multi-unit clusters with middle to high BFs. In some cases, correct classification is higher than would be expected from the relative spectral content (“purr,” “chutter short,” “tooth chatter”). The error was computed via error propagation and is the standard error of the mean. (C) Relative frequency content for the 100 ms segment of each vocalization which elicited the responses used for the classification. The main frequency contents of the vocalizations are spread across the entire analyzed BF frequency range.

**Figure 8:**
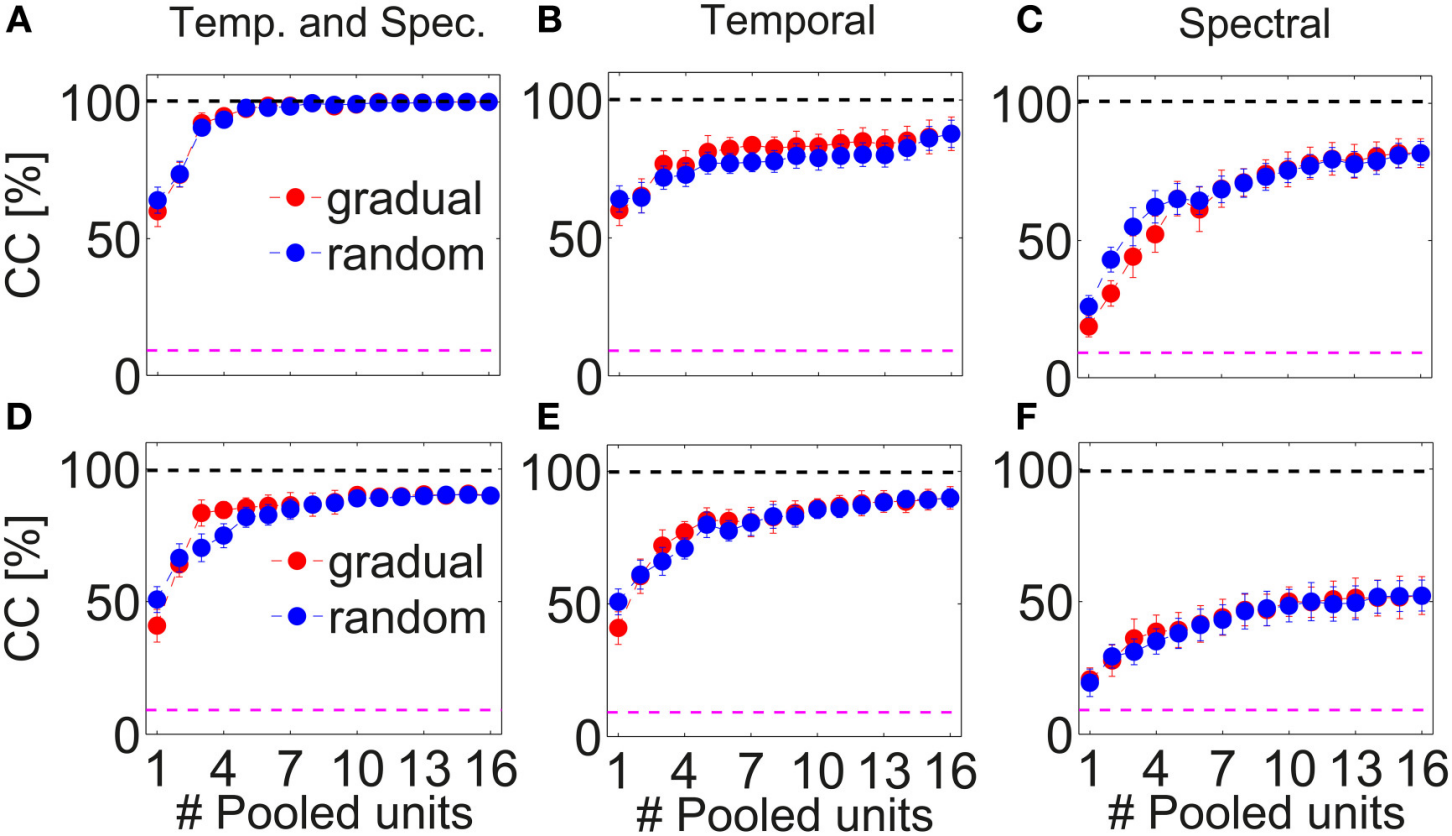
**Pooling responses from several multi-unit clusters**. Correct classification of spiking responses to 11 different vocalizations for an increasing number of pooled multi-unit clusters. Spiking responses are concatenated or added to preserve (A,D) spectral and temporal; (B,E) only temporal; (C,F) only spectral response information. Pooling of responses significantly increases correct classification. Multi-unit responses were pooled either from a recording across the best frequency gradient (A–C, with BF = 0.5–23 kHz) or from a tetrode-recording yielding similar frequency tuning of the recorded multi-unit clusters (D,E, BF = 6.3–8 kHz), and were taken, respectively, from simultaneous recordings of one shank. Perfect classification was achieved when using temporal and spectral information across laminae (A) and nearly perfect classification was achieved when using temporal and spectral information and also when using only temporal information (D,E). Multi-unit clusters to be successively pooled were selected either gradually in tuning preferences (_
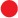
_) or chosen randomly(_
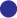
_). Averages over 2–3 iterations are shown. Gradually and randomly pooled responses yield very similar values of correct classification. The dashed pink line denotes chance level, the top black dashed line is drawn at 100% correct classification.

**Figure 9:**
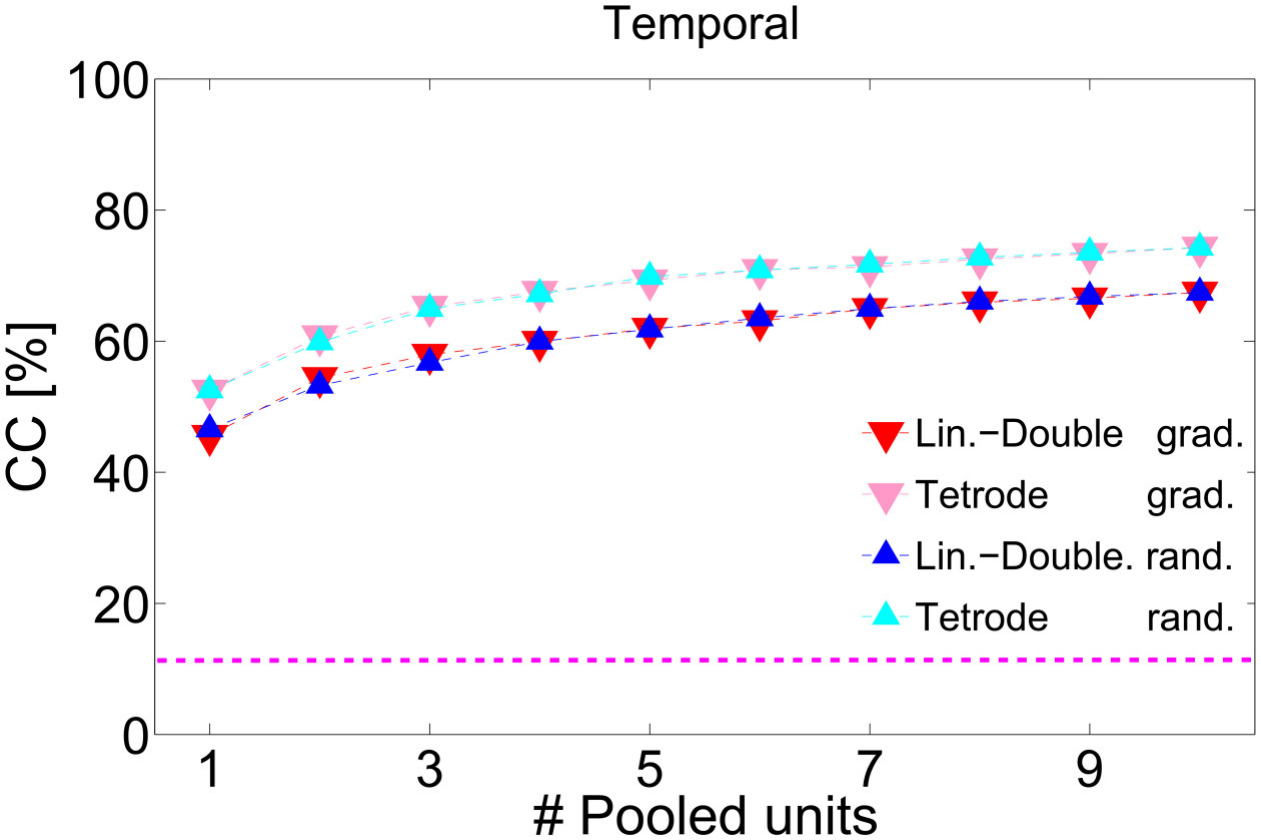
**Average correct classification across all recordings**. Correct classification for pooling temporal information of spiking responses. Correct classification is higher for pooling multi-unit clusters from with similar frequency tuning (tetrode-recording), than along the best frequency gradient (linear-Double-shank recording). Gradual (_
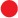
_,_
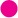
_) and random (_
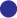
_,_
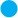
_) pooling yield similar correct classification values, for similarly frequency-tuned unit and across the frequency gradient. The dashed pink line denotes chance level.

**Figure 10:**
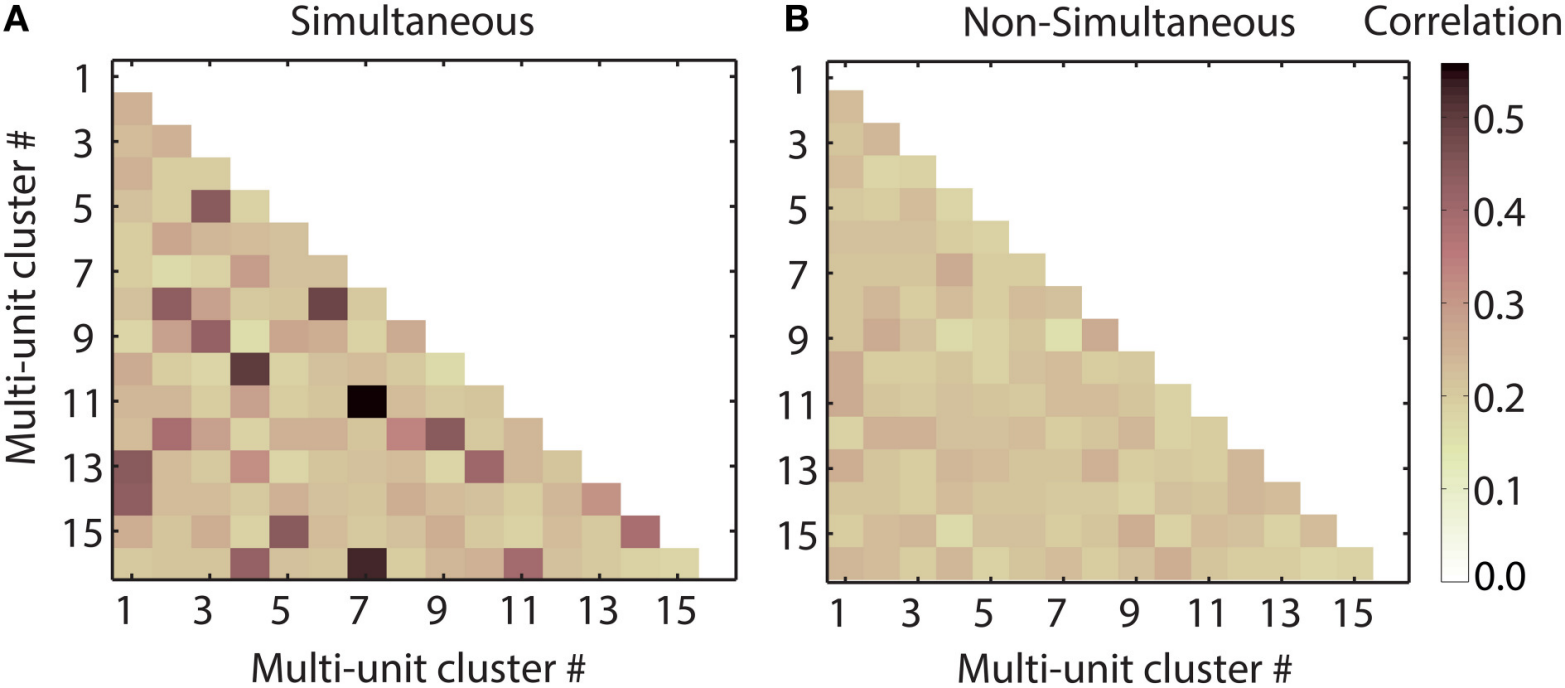
**Temporal response correlations of multi-units clusters**. Half-matrices of coefficients of correlation (within a maximum possible delay of 10 ms) between responses recorded simultaneously (A) and non-simultaneously (B), in response to the “squeal” presented at 70 dB SPL, from 16 multi-units of one shank spanning a best frequency range of 0.7–22.6 kHz. For simultaneous responses, correlation values differ across multi-unit pairs. Some multi-unit pairs display higher correlation for simultaneous responses than for non-simultaneous ones. Other multi-unit pairs show similar values for simultaneous and non-simultaneous responses. The correlation matrices are symmetric and their diagonal is unity, hence only their lower halves are displayed.

**Figure 11:**
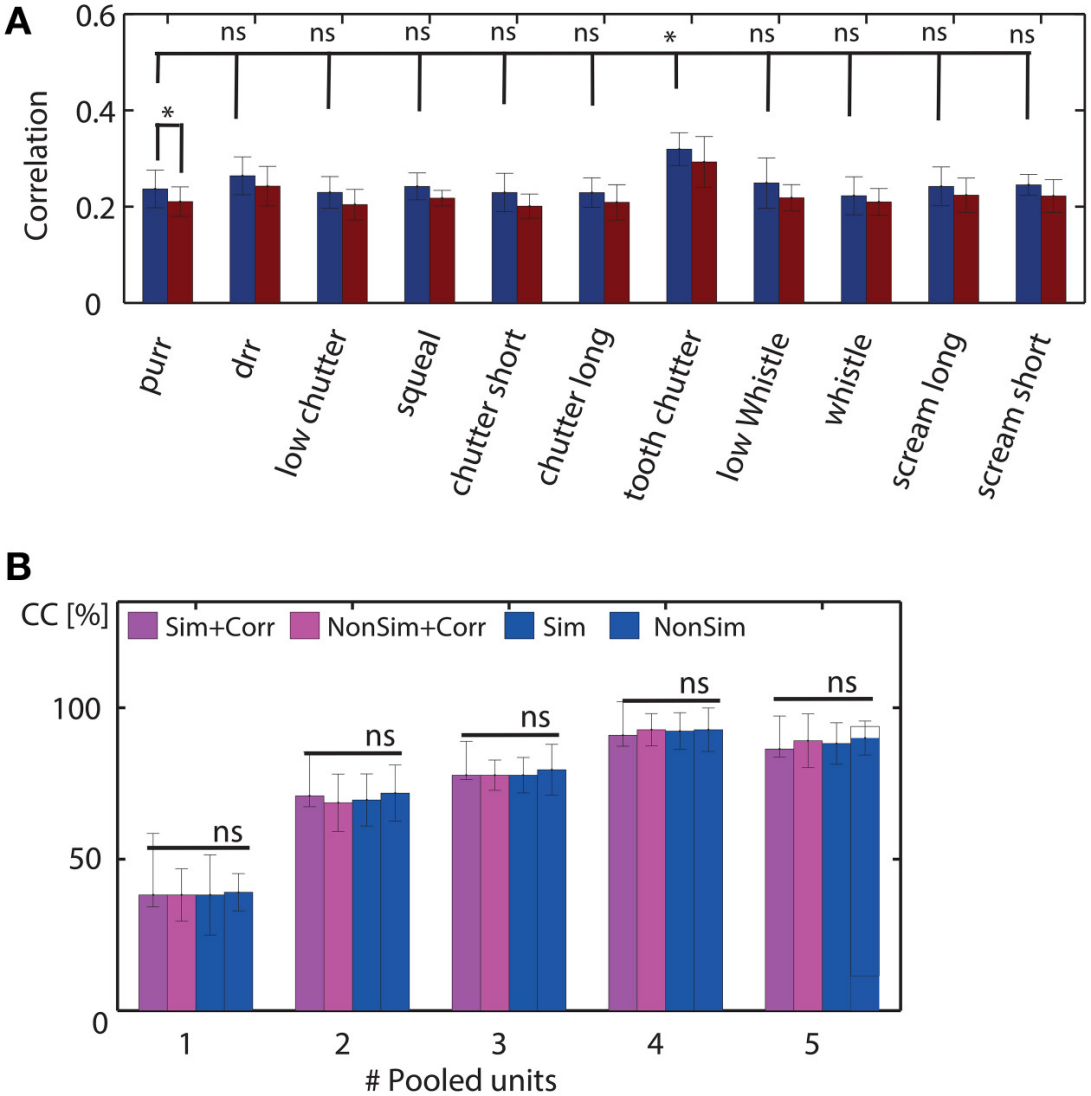
**Temporal response correlations of combined multi-unit clusters and their effect on neural discrimination. (A)** Average correlations of all (*n*_comb_ = 5) pooled multi-unit clusters for each vocalization are compared for simultaneously and non-simultaneously recorded responses, for a stimulus intensity of 70 dB SPL. Simultaneous and non-simultaneous response correlations differ significantly for each vocalization (example displayed for “purr”). Correlation values across vocalizations do not vary significantly, except for responses to the “tooth chatter” which are significantly higher than response correlations elicited by all other vocalizations. (B) Comparison of correct classification between all 11 vocalizations for pooling 1–5 responses recorded simultaneously and non-simultaneously, for explicitly including response correlations (Sim+Corr, NonSim+Corr) and when not including them (Sim, NonSim), at 70 dB SPL. Pooling increases neural discrimination as shown previously. Simultaneity of recordings does not produce a detectable difference in correct classification (^*^ denotes significant results, ns non-significant; mean, *p* = 0.05, Student's *t*-test).

Classification was tested for several data types, features and classifiers, and classification performances for these three parameters are compared, see Figure 3. The approach using the correlation of spike trains was then employed for all subsequent analyses (Figures 4–**11**). The classification yields the percentage of correctly assigned responses to each stimulus (Correct Classification, *CC* [%]). A confusion matrix (*Conf*) gives the correct and false classifications, rows representing assigned stimulus classes, columns representing the actual presented stimulus classes. Perfect classification would only yield entries on the diagonal. The entries of the confusion matrix on the diagonal represent the correct assignments for each individual vocalization *k* = 1, …, *N*_voc_, averaged across all cross-validation iterations *N*_xval_.

(2) $$\begin{array}{l} CC\left(k\right)=\frac{1}{N_{\text{xval}}}\overset{N_{\text{xval}}}{\underset{x=1}{\sum }}Conf_{x}\left(k,k\right) \end{array}$$

The mean across the diagonal, averaged over all cross-validation iterations, yields the correct classification between the *N*_xval_ = 11 vocalizations.

(3) $$\begin{array}{l} CC=\frac{1}{N_{\text{voc}}}\overset{N_{\text{voc}}}{\underset{k=1}{\sum }}CC\left(k\right) \end{array}$$

The classification error is the standard deviation obtained from all cross-validation steps. To obtain the best discriminative length and location of the spike trains within the whole recording, classification for different segments was compared. Classification between all 11 vocalizations was computed for consecutive non-overlapping 10 ms segments of the spike train, starting at the onset of the recording. This is aimed to compare the discriminative information contained within short spike train windows before and after stimulus onset. Classification of spike trains of length *l* (*l* = 5, 10…50, 100…900 ms), was computed in order to test which length is sufficient for high discrimination values. The starting point was chosen to be over 70 ms after recording onset, since all vocalizations have clearly begun after this time, see Figure 1. The vocalizations display amplitude modulations and frequency content changes over time. Do some segments of the vocalizations yield better discrimination between all 11 vocalizations than others? To test this, we compared classification accuracy for consecutive non-overlapping spike train segments, starting from 70 ms after recording onset. The segments were chosen to be 100 ms long, the temporal window which was used subsequently for the pooling analysis.

The vocalization-specific classification compares how well a spike train response to a specific vocalization can be discriminated against responses to all other 10 vocalizations, of which some are quite similar (e.g., “short scream” and “long scream”). It allows one to make inferences about how detailed this vocalization is encoded with respect to other vocalizations. The approach not only comprises a representation of the temporal spiking response across differently frequency-tuned neurons to a given vocalization (corresponding to a neurogram), but furthermore compares the spike trains of differently tuned neurons, and compares these representations across vocalizations.

### 2.5. Pooling spiking responses from different multi-unit clusters

To assess whether combining responses from several multi-unit clusters improves discrimination between stimuli, simultaneously recorded responses from different multi-units to the same stimulus were pooled. Note that the use of multi-unit clusters for the study could limit the ability to assess the degree to which pooled correlated firing may encode temporal features in the vocalizations.

Figure 2B illustrates the procedure for pooling responses from three multi-unit clusters with different best frequencies. In this example, two vocalization stimuli are displayed, therefore two responses combined from three multi-unit clusters to each vocalization (stimulus 1 or 2) are classified. Pooling methods have been applied before, and are discussed in more detail in Schneider and Woolley (2010). Schneider and Woolley added single neuron spike trains in zebra finches exposed to songs. In our work, spike trains obtained from simultaneous recordings of different multi-unit clusters were either added or alternatively concatenated. Concatenation preserves the frequency information because the order in which responses from multi-unit clusters are concatenated is the same for all combined responses that are compared. Thus, during classification, spike trains from the same multi-unit clusters with their specific frequency tuning, but in response to different vocalizations, are compared. Concatenation preserves the temporal spiking response separately for each multi-unit cluster. For recordings from one shank with a double-tetrode array, several multi-unit clusters have similar frequency tuning. They have similar best frequencies, but might have different preferences for amplitude modulations (AM) depending on their spatial distance within the ICC (Schreiner and Langner, 1988; Baumann et al., 2011). Therefore, in this case, concatenation additionally preserves information of amplitude modulation preferences. Addition of spike trains maintains temporal information.

However, as responses from differently tuned multi-unit clusters are added and are not distinguishable anymore, the spectral information is lost. Multi-unit clusters were combined in a third manner to preserve only spectral information. For this purpose, mean firing rates for each multi-unit cluster and trial were computed from spike trains of 100 ms duration. These firing rates were then concatenated in the same way as the spike trains had been concatenated, as mentioned above. By doing this, spectral information is preserved but temporal information is lost. These feature vectors were assigned to the class which yielded the minimum squared difference in firing rate of test and training trial.

Neural discrimination was computed for a successively increasing number of pooled multi-unit clusters. The additional multi-unit cluster to be pooled was either the nearest neighbor, or alternatively was chosen at random. In the first case, information from similarly tuned multi-unit clusters is successively combined, whereas in the second case, information from units with very different tuning is combined. Performance values are averaged over two iterations for gradual pooling and over three iterations for random pooling.

### 2.6. Canceling temporal correlations when pooling responses

Multi-unit clusters may interact with one another in order to more efficiently encode complex sounds. This neural interaction can lead to temporal correlations of their responses. These temporal correlations of the multi-unit responses are only present in recordings which were acquired simultaneously. To test if the temporal correlations have an effect on the encoding, we compared the neural discrimination when they were present and when they were absent. Hence, in order to cancel the temporal correlations, simultaneously recorded trials of different multi-unit clusters were randomly shuffled (Abeles, 1982) before combining and comparing them. Correlations of the responses and classification when explicitly including these correlations were compared for simultaneous and non-simultaneous responses. Correlations between responses for combining *n*_comb_ multi-unit clusters, *CorrRes*(τ), were computed in the following manner: pairwise, spiking responses from two multi-units *x*(*t*), *y*(*t*) of length *n*, were cross-correlated and the highest value within a maximum possible delay of τ between the responses was selected

(4) $$\begin{array}{l} CorrRes\left(\tau \right)=\text{max}\left(\frac{\sum_{t=1}^{n-\tau }\left(x\left(t+\tau \right)-\langle x\rangle \right)\cdot \left(y\left(t\right)-\langle y\rangle \right)}{\sqrt{\sum_{t=1}^{n}{\left(x\left(t\right)-\langle x\rangle \right)}^{2}\sum_{t=1}^{n}{\left(y\left(t\right)-\langle y\rangle \right)}^{2}}}\right) \end{array}$$

for τ = [−10 *ms*, 10 *ms*] (see **Figure 10**). This delay is within the range of maximum response latencies in the ICC (Langner et al., 1987). The average correlation value between all combined multi-unit responses, for *n*_trial_ trials, was then compared for simultaneous and non-simultaneous recordings, see **Figure 11A**. The average correlation value for each multi-unit cluster with all other multi-unit clusters within the combined set, *n*_comb_ correlation values, were included in the classification procedure (**Figure 11B**). Test trials were assigned to the class for which the training and test trials had, on average, maximum correlation of the time courses, *Corr*, as described in Section 2.4, and minimum average squared differences of response correlations, *CorrRes*. Significance was assessed using the Student's *t*-test for normal distributions, and the Wilcoxon-Mann-Whitney test for comparison of non-normal distributions.

### 2.7. Averaging across multi-unit clusters

One goal of this work was to study neural discrimination of individual vocalizations with their specific spectrotemporal properties across multi-unit clusters with different frequency-tunings. However, average neural discrimination between all behaviorally relevant vocalizations—a representative set was used for this work—is of interest, since this is the task that the auditory system of the guinea pigs solves perfectly, allowing the guinea pigs to react to these communication calls (Berryman, 1976). We are aware that we might be averaging out preferences for specific vocalizations. Recordings were acquired from 11 guinea pigs in 3–4 electrode insertion positions (taken altogether 36 positions), with activity recorded simultaneously from 32 recording sites, the multi-unit clusters. This yielded 72 sets of simultaneously recorded activity from 16 multi-unit clusters from one shank.

In this work, in order to avoid smearing out differences in discrimination (see Section 3.3), rather than averaging across all 1152 multi-unit clusters, the recordings from one shank (16 multi-units clusters) have been classified for one analysis, and the analysis has been repeated for all 72 shanks. It was verified that the observed trend is consistent across all shanks. Discrimination is compared for individual or pooled multi-unit clusters of one shank of one recording in **Figures 6, 8, 11**. Findings were verified to be generally true for all such sets. But averages across these sets are also given (**Figures 7, 9**).

We tested classification accuracy across different animals (Figure 5). When averaging across multi-unit clusters, electrode positions or animals, the classification error was computed via error propagation. The range of best frequencies from the recorded multi-unit clusters varied for each animal. Therefore, the number of multi-unit clusters, for which averages were taken for one best frequency (Figures 5B, **7A,B**), varied across animals. Differences in neural discrimination based on different amplitude modulation preferences would be averaged out when taking the mean across multi-unit clusters with the same best frequency.

In the first part of the analysis a sufficient classification procedure and optimal response length and location within the recording are selected (Figures 3, 4). For this purpose one shank-recording set with overall high classification performance and which covers a wide range of best frequencies was used.

## 3. Results

We analyzed neural discrimination between a spectrotemporally rich set of 11 species-specific vocalizations for 1152 multi-unit clusters across the central inferior colliculus of 11 guinea pigs. Using neural discrimination we tested variation of encoding accuracy of individual vocalizations across the best frequency gradient of the ICC. We then combined spike train responses from several multi-units to investigate whether groups of multi-unit clusters result in a better neural discrimination than one multi-unit cluster, and whether temporal response correlations between the multi-unit clusters contribute to an even better separability. In a preliminary analysis we selected a classification procedure and response length which yielded the best classification performance for these multi-unit responses. Additionally, we compared the averaged classification performances across animals and best frequencies.

### 3.1. Correlation of EPSP-spike trains yields best classification performance

To determine which data type (EPSP-spiking response or local field potential), feature of the response and classifier yield the maximum correct classification for neural discrimination between the 11 vocalizations, we compared the performance for different combinations of data type, feature, and classifier on responses to 300 ms segments of the vocalizations. The focus of our study was the separability between multi-unit responses from the central inferior colliculus, the output, thus the spiking activity and not the synaptic input, which is captured by the local field potential (Pettersen et al., 2012). Hence, we used spiking activity for the analyses. Nevertheless, classification performances for local field potential responses are also compared. Performances have been tested for sets of multi-unit cluster. Figure 3 displays an example of the classification performance for recordings from one shank. The performances were averaged across 10 multi-unit clusters from one recording (Figure 3, top), and are also shown for the individual multi-unit clusters with different best frequencies (Figure 3, bottom). The best frequencies span a range between 0.5 and 18 kHz. Using correlation to classify 300 ms long EPSP-spike trains yielded the highest correct classification (85%). But differences to some of the other combinations were minor. The naive Bayesian classifier and linear discriminant analysis on the 6-dimensional feature vector with firing rates across 50 ms also yielded high correct classification values (75%). However, a specific focus of this study was to compare the finer temporal structure of the multi-unit responses (below 10 ms) which has been shown to be crucial for neural discrimination (Schnupp et al., 2006). This is achieved by the correlation approach which takes into account a 3 ms-resolved temporal structure. Classification performances for discrimination between 11 vocalizations varied for multi-unit clusters with different best frequencies. However, maximum correct classification values did not systematically correlate with the best frequency of the multi-unit cluster (Figure 5B).

Using the correlation-based approach for discrimination of spike trains provides the advantage of comparing the temporal structure of responses and it achieves the highest degree of accuracy for classification performance as defined in the Materials and Methods, Section 2.4. Therefore, the method using the correlation of spike trains was employed for the subsequent analyses.

### 3.2. Separability of spike trains does not vary with starting point

In order to test from which starting point within the 1 s recordings and which length of spike trains should be employed for the analyses, we compared the correct classification between all 11 vocalizations for: (1) consecutive spike trains of 10 ms before and after stimulus onset, (2) spike trains of (5, 10–50, 100–900) ms duration, and for (3) 100 ms long consecutive spike trains covering the whole recording time of 1 s. The starting point for analysis (2) was chosen to be at least 40 ms after the vocalizations had started (Zheng and Escabí, 2008), as we are not investigating onset responses in this study, and was also chosen not to fall into a segment of the vocalization which provided no characteristic information (e.g., segment 0.7–0.9 s for the “low chutter,” Figure 1). Due to the vocalizations being diverse, these starting points varied across vocalizations, but were kept constant, respectively. The starting points were, respectively, 0.54, 0.53, 0.54, 0.11, 0.52, 0.53, 0.06, 0.09, 0.53, 0.53, and 0.21 ms in the order of the vocalizations displayed in Figure 1. The analysis was performed on multi-unit clusters from one recording covering a BF-range of 0.63–20.1 kHz, and the average value of all 16 multi-unit clusters was used for display (Figure 4).

(1) Figure 4 displays the percentage correct classification of 10 ms-long spike trains. Before stimulus onset, classification performance is at chance level (1∕11≈ 9.1%) and increases gradually after stimulus onset reaching 25 ± 5% at about 50 ms after stimulus onset (which is 70 ms recording time).

The increase after stimulus onset is gradual and not sharp because the actual starting times of the individual vocalizations differ (e.g., Figures 1A,B). After about 70 ms recording time, classification does not increase further, hence no additional discriminative information is present in the responses; vocalizations have begun at this time (Figures 1A,B). Performance values are relatively low due to the short spike train length of only 10 ms.

(2) Classification accuracy gradually increased for increasing spike train length. Performances were, respectively, 19 ± 2%, 55 ± 4%, 83 ± 4%, *and* 94 ± 3%, for lengths of 5, 100, 300, and 900 ms. This increase of correct classification with spike train length was shown for recordings in zebra finches and grasshoppers (Wohlgemuth and Ronacher, 2007; Schneider and Woolley, 2010). Machens et al. (2003) found spike train lengths above 400 ms to yield near to perfect neural discrimination.

(3) Classification performance for consecutive 100 ms long spike trains across the recording of 1000 ms did not vary significantly, ranging from 53 ± 3% to 58 ± 3%. The exact starting point of these spike trains within the 1 s recording was irrelevant. For the rest of the study, the starting points of analysis (2) were employed consistently.

To analyze classification performance across animals and positions, 300 ms long spike trains were used, since these include the responses to all ongoing 11 vocalizations (Figure 5). In order to increase difficulty of neural discrimination and more clearly see differences in performance, for the analysis of individual vocalizations in relation to the best frequency of the the multi-unit cluster, and when pooling responses of several multi-unit clusters, 100 ms spike trains were used (Figures 6–**8, 10, 11**).

### 3.3. Discrimination varies across animals and electrode insertion locations, and increases with stimulus intensity

In order to test the validity of averaging classification values across all recordings, we compared correct classification between 11 vocalizations of 300 ms long spike trains, across average classification values from each of the 11 animals. We also compared classification values for different insertion locations in one animal (average across 32 multi-unit clusters), and investigated how values vary with the intensity at which the vocalizations were presented, from 30 to 70 dB SPL in steps of 10 dB SPL.

Correct classification varies significantly across animals (see Figure 5A), the best discrimination being 85% for animal 1 (Experiment 1). Performances of some animals are very similar (e.g., Experiment 1 and 2). Classification values vary across different electrode insertion locations P (e.g., P_1_ = 81.1 ± 1.5%, P_2_ = 71.6 ± 1.4%, P_3_ = 70.0 ± 1.2%, P_4_ = 67.2 ± 1.2% at 70 dB SPL). The overall classification performance increases with stimulus intensity (P_30dB_ = 22.2 ± 1.2%, P_40dB_ = 40.3 ± 1.4%, P_50dB_ = 60.1 ± 17%, P_60dB_ = 73.9 ± 1.8%, P_70dB_ = 81.1 ± 1.5% for P_1_), as firing rates increase for higher intensities. Figure 5A displays the average correct classification for each animal and each best frequency. Responses from some animals are overall better discriminated than others (e.g.,# 2), however, correct classification does not depend on the multi-unit's best frequency and varies across animals.

Thus, in order to avoid smearing out differences in discrimination, in the subsequent analyses, classification values were not averaged across animals and electrode positions but taken from the recordings of one shank of one animal. This was repeated for respectively all 72 shanks, and results for example recordings of one shank are given. Recordings for stimulus intensities at 70 dB SPL were employed for our analyses, as they yield the highest classification performance.

### 3.4. Discrimination of individual vocalizations depends on best frequency

The total neural discrimination between responses to all vocalizations does not vary systematically with the multi-unit cluster's best frequency (BF), as shown above. However, neural discrimination of individual vocalizations might differ with best frequency, depending on their spectral content. To address this question we compared discrimination of 100 ms-long spike train responses to four vocalizations for several multi-unit clusters with different best frequencies.

A relatively short spike train length of 100 ms was chosen in order to raise the separability threshold and enable the detection of subtle differences in discrimination. The four vocalizations were divided into two groups; vocalizations of the same group have similar spectral content. Figure 6 shows correct classification between responses to the “drr,” “low chutter,” “whistle” and “long scream.” These have main spectral energy below 1.5 kHz (“drr,” “low chutter,”Figures 1C,D), thus low frequency content, or main frequency contents above 1.5–2 kHz (“whistle,” “long scream,” Figures 1G,I), a broad spectrum of frequencies.

Responses were recorded with a tetrode-array (Figure 6A) and a linear double-shank array (Figure 6B), from multi-unit clusters with various best frequencies. Responses of middle-BF multi-unit clusters to the two vocalizations which contain a broad spectrum of frequencies are perfectly discriminated, whereas the vocalizations with low frequency content are poorly discriminated (Figure 6A). Using low-BF multi-unit responses, the vocalizations with low frequency content are nearly perfectly discriminated (Figure 6B). A clear transition toward a perfect discrimination of vocalizations containing middle and high frequencies is visible as the multi-units' BFs increase. This clear preference for discriminating certain groups of vocalizations over others was also observed for different combinations, e.g., “drr,” “low chutter,” “squeal,” and “low whistle.” The performances of these four individual vocalizations for classification between all 11 vocalizations, averaged across all 1152 multi-units are displayed in Figure 7A, for BFs between 0.5 and 25.4 kHz. Classification values from multi-unit clusters with the same best frequency showed a similar frequency dependence and were averaged to give only one value per best frequency. A trend of higher correct classification of vocalizations containing low frequencies from responses of low-BF multi-unit clusters, and of vocalizations with broad spectral distributions from responses of middle-and high-BF multi-unit clusters is apparent. Figure 7B shows the performances for the remaining seven vocalizations for classification between all 11 vocalizations, averaged across all 1152 multi-unit clusters. Also displayed are relative frequency contents for these four vocalizations which are plotted separately for all 11 vocalizations in Figure 7C. These show a similar trend to the classification performances, and for some frequencies are very similar to the classification performances (e.g., “scream long”). Deviations exist (e.g., “purr”) and could be attributed to preferred encoding of fast amplitude modulations by low BF neurons (Rodriguez et al., 2010), and hence a better discrimination of vocalizations containing fast amplitude modulations than would be predicted solely by their frequency content.

The match between the averaged correct classification and relative frequency content for 10 vocalizations varied between 74–80%, using Euclidean distance. The “tooth chatter” displayed only a match of 53%. Trends of preferred encoding for the individual vocalizations along the BF gradient exist. The “tooth chatter,” and “chutter short” do not show pronounced preferred encoding by certain best frequency multi-unit clusters. The “purr” displays bimodally distributed preferred encoding. Thus, with the exception of two vocalizations, optimal discrimination of individual vocalizations is spatially distributed across the ICC. Figure 7C illustrates the relative frequency content for the 100 ms segment of each vocalization which elicited the responses that were used for the classification. Figure 1 displays spectral contents for the entirety of each vocalization. Whereas the “purr,” “drr,” and “low chutter” have main spectral contents at low frequencies, the “whistle,” “long scream,” and “short scream” have almost no energy at these frequency ranges, but have important relative power at frequencies above 2 kHz. Main spectral contents of the 100 ms vocalization segments are distributed across the whole BF-range.

Accurate encoding of a vocalization in the spiking response allows good discrimination against other vocalizations. Perfect discrimination between responses to very similar vocalizations indicates that these vocalizations are encoded in detail and hence can be separated based on minor spectrotemporal differences. If, on the other hand, vocalizations are coarsely encoded, responses to similar vocalizations are frequently confused. The “tooth chatter” and “long chutter” display important frequency content across the whole range of best frequencies (see Figure 1), thus they are not preferentially encoded by a certain subset of best frequency multi-unit clusters.

Vocalizations with low frequency contents are preferentially encoded by low-BF multi-unit clusters and vocalizations with middle-high frequency contents are preferentially encoded by middle-high BF multi-unit clusters. Optimal encoding of individual vocalizations is broadly distributed across the tonotopy. Main spectral contents of the vocalizations also display a spread arrangement across the BF-range.

### 3.5. Pooling multi-unit cluster responses

To test whether the combined response of several multi-unit clusters yields better neural discrimination of all 11 vocalizations, we pooled 100 ms-long spike trains from a successively increasing number of multi-unit clusters by either concatenating or adding the spike trains. Concatenation preserved the spectral and temporal information whereas addition only preserved the temporal information. Additionally, we performed neural discrimination of the average firing rates of the pooled multi-unit cluster. Concatenating the units' firing rates preserved only spectral information. To test if pooling spectral and temporal information effects neural discrimination in a similar way, we compared the three cases: pooling only spectral, only temporal, or spectral and temporal information.

We pooled multi-unit cluster from a range of differently frequency-tuned multi-unit clusters from along the BF-gradient (linear-double-shank recording) and we also pooled multi-unit cluster with similar frequency tuning (double-tetrode recording). Do differently tuned multi-unit cluster (in frequency or amplitude modulation) yield more information than similarly tuned ones when being combined? To answer this question, responses were pooled in two different sequences. The additional multi-unit to be pooled was either the nearest spatial neighbor or was chosen randomly. Whereas in the first case, one successively combines information from similarly tuned units, gradually increasing difference in tuning, the second case allows us to combine information from units with very different frequency tuning (or amplitude modulation preferences).

Combining responses from several multi-unit clusters significantly increases discrimination. Figure 8 depicts correct classification for pooling information from successively 1–16 multi-unit clusters along the BF-gradient (example data from a double-shank recording from one shank, Figures 8A–C) and responses from multi-unit clusters of similar frequency tuning (example data from a double-tetrode recording from one shank, Figures 8D–F). Figure 8A demonstrates that correct classification continuously increased significantly when combining temporal and spectral information of up to five multi-unit clusters. Combining five multi-unit clusters led to perfect discrimination, and was not degraded by combining further multi-units. Although the individual courses of correct classification varied across experiments, classification accuracy always increased for up to 3–6 multi-unit clusters before reaching saturation. These combined responses contain about 9–30 single neurons and additional background noise. Pooling yielded near to perfect (on average 90%) correct classification for all recordings, and several cases had perfect accuracy. Thus, pooling short-time responses of 3–6 multi-unit clusters is sufficient to uniquely represent 11 different vocalizations.

The increase in correct classification when combining an additional multi-unit cluster was higher when using both, temporal and spectral information (10.2 ± 1.5% per unit) than when using only temporal information (5.2 ± 1.6% per unit), Figures 8A,B. For the latter case, correct classification did not increase significantly when pooling more than five multi-units, but stayed constant at around a value of 80%. Correct classification of firing rates was very low for one multi-unit cluster (20%), increased for pooling up to five multi-unit cluster (8% per unit), then continued to increase at a lower rate (2% per unit) up to a performance level of 80% and stayed constant for pooling more than 10 multi-unit cluster (Figure 8C). Using only temporal spiking information, although saturating at a low number of pooled multi-unit cluster, will not recover the entire information about the encoded signal. Using only spectral information will also not recover the entire encoded information, and a much larger number of units needs to be pooled to achieve comparable correct classification. Thus, for near to perfect discrimination, spectral and temporal spiking information are necessary.

The overall increase in correct classification did not significantly differ when pooling multi-unit cluster, either gradually or randomly. Note that the nearest neighbor in the case of linear double-shank recordings is 100 μm away, mostly in the direction along the best frequency gradient (see Materials and Methods, Section 2.1). (The higher correct classification for gradual pooling than for random pooling of 3–4 multi-unit clusters in Figure 8D is present in this recording set but not systematically across sets, because the pooled multi-unit cluster sets from different shanks display different classification courses). This suggests that pooled responses from very differently tuned (in frequency) as well as from more similarly tuned multi-unit clusters yield a better discrimination performance than the response from one multi-unit cluster. Not only large differences in tuning and in responses but also relatively small differences provide new information to uniquely represent vocalizations in the ICC.

The above stated results are generally valid when pooling responses of multi-unit clusters from a tetrode recording (Figures 8D–F). However, there are the following exceptions: the courses of classification for using both, temporal and spectral information, are similar to using only temporal spiking response information, and both cases reach a maximum performance of 90%. Still, more units are needed to reach this maximum value when using only temporal spiking response information. Pooling spectral information yields a maximum performance of only 50%. In this case, responses from similarly frequency-tuned multi-unit clusters do not contain enough information to perfectly discriminate between all vocalizations. Using only temporal information is sufficient to nearly perfectly represent vocalizations. Thus, mostly temporal information is encoded by multi-unit cluster sets of similar frequency tuning.

The average of all 72 pooling procedures confirms the above stated result (Figure 9). Temporal information is encoded in more detail by combined responses from multi-unit clusters with similar frequency tuning than from multi-unit clusters across a broader range of the best frequency gradient and thus leads to a higher correct classification. Correct classification is almost identical for gradually and randomly pooled responses.

### 3.6. Temporal response correlations do not enhance neural discrimination

Simultaneously responding multi-unit clusters can interact in order to more efficiently encode vocalizations. This interaction can lead to temporal correlations of their responses. The correlations between the multi-unit responses could improve separability in several ways. One possibility might be that the interaction strength between the multi-unit clusters and, consequently, their temporal correlations varies for responses to different vocalizations and thus aids discrimination between the vocalizations. Another possibility would be that the multi-unit clusters' simultaneous responses are more similar to each other than non-simultaneous ones, hence their temporal correlations are stronger and by means of redundancy, vocalizations are more faithfully represented. We tested these possibilities by comparing the averaged correlation values between all multi-unit responses of one pooling, to each vocalization, for simultaneously and non-simultaneously recorded responses.

We combined multi-unit clusters and their averaged correlation values by concatenation, since this approach preserves temporal and spectral information and yielded higher correct classification values than addition of responses, as stated above. Pooling of non-simultaneously recorded responses was performed by randomly shuffling the 20 trials of each multi-unit response before combining the responses. Figure 10 shows the pairwise correlation of spiking responses for all 16 multi-unit clusters of one recording, for one vocalization. Correlations of simultaneously recorded responses are higher than or as high as correlations of non-simultaneous responses. The correlations vary for different pairs of multi-units clusters. In Figure 11A, the averaged correlations for simultaneously and non-simultaneously recorded responses between multi-unit clusters are displayed for each vocalization. For each vocalization, correlation values of simultaneously recorded responses are significantly higher than those from non-simultaneously recorded ones (mean, *p* = 0.05, Student's *t*-test). Correlations do not vary significantly across vocalizations, for neither simultaneous nor non-simultaneous responses. The only exceptions are responses to the “tooth chatter,” which yield significantly higher correlations than responses to other vocalizations. Responses to the “tooth chatter” are phase-locked to the envelope for multi-unit clusters across all studied best frequencies. This leads to higher correlation values of those responses. However, these significantly higher correlation values do not yield significantly higher classification values for the “tooth chatter,” but are comparable to other vocalizations such as the “long scream.” Thus, should temporal correlations facilitate a more unique neural representation of vocalizations, then this might be achieved via redundancy.

If temporal correlations promote a unique representation of the vocalizations and hence a better neural discrimination, then their cancelation will lead to a decrease in neural discrimination. We tested the hypothesis that temporal correlations improve separability of responses by comparing neural discrimination when pooling simultaneously or non-simultaneously recorded 100 ms long spike trains. We explicitly included response correlation values between the multi-unit clusters of one pooled set in the feature vector. However, we also compared classification values of pooled responses for which response correlations were not explicitly included.

In Figure 11B, correct classification between all 11 vocalizations is compared for pooled simultaneously and non-simultaneously recorded responses. Correct classification does not vary significantly for pooling simultaneous or non-simultaneous responses neither for the case of including the multi-unit clusters' temporal response correlations nor for the case of not including them. Correct classification increased when pooling additional multi-unit clusters, as described above, and did not increase further when pooling more than five multi-unit clusters. To increase the complexity of neural discrimination, i.e., to raise the threshold, we compared correct classification for pooled simultaneous and non-simultaneous responses for stimulus intensity levels of 30–70 dB SPL, in steps of 10 dB SPL. Overall, correct classification values were lower, however, classification did not differ significantly between simultaneous and non-simultaneous pooled responses. Response correlations were computed, allowing for a delay between the responses from different multi-unit clusters. If no delay is assumed, the computed non-simultaneous response correlations are much lower than simultaneous ones, and classification performance is significantly higher for non-simultaneous recordings than for simultaneous ones. Thus, in this case, temporal correlations are found to be detrimental for neural discrimination (mean, *p* = 0.05, Student's *t*-test).

We conclude that temporal response correlations from different multi-unit clusters do not promote a unique representation of the vocalizations. Hence, combining responses from several multi-unit clusters improves classification, but this is not because temporal correlations between simultaneously responding units contribute to the improvement.

## 4. Discussion

We found that vocalizations in the mammalian inferior colliculus are encoded spatially across the best frequency gradient of the inferior colliculus. A small number of independent multi-unit clusters are often sufficient to reliably encode the representative set of behaviorally relevant vocalizations.

In the mammalian ICC, discrimination of vocalizations has previously been studied based on spike-rate (Portfors et al., 2009). Spike-timing information is crucial, though, for neural discrimination of vocalizations and intelligibility of speech (Shannon et al., 1995; Schnupp et al., 2006). Here, we used spike trains, preserving their timing information, to perform neural discrimination across 11 vocalizations, and investigate encoding of individual vocalizations for differently frequency-tuned multi-unit clusters. Our findings are based on a large set of multi-unit clusters (*N* = 1152), of which the best frequencies span a range between 0.5 and 45 kHz. The studied vocalizations are a representative set of behaviorally relevant stimuli (Berryman, 1976) and it has been suggested that neurons are adapted to encode them (Rieke et al., 1995). Thus, these natural stimuli are well suited for studying the neural encoding of sounds in the midbrain. The encoding of vocalizations by combined simultaneous responses and the impact of temporal correlations between those responses has not been investigated previously in the mammalian ICC. Since anesthesia has non-negligible effects on the neural activity (Astl et al., 1996), neural discrimination, especially for units that did not yield perfect classification for pooling, is likely to improve in awake animals. Still, the neural discrimination values obtained in our study were very high, in several cases even perfect.

### 4.1. Optimal encoding of individual vocalizations

Encoding averaged across several vocalizations does not vary systematically with best frequency (Schneider and Woolley, 2010). However, we showed that optimal encoding of individual vocalizations depends on the best frequency of the neurons. Deviations from a linear spectral mapping exist, and are interesting for future studies. One central observation is that, in general, frequency ranges with high discriminative power are rather broad. Thus, in spite of the finding that main spectral contents of the studied vocalization segments display a wide distribution across frequencies (Figure 7C), discriminability remains very good and this should be beneficial in a behavioral context.

Preferred encoding along the BF-gradient follows, in most cases, a similar trend to the relative spectral content of the vocalization, such that vocalizations which contain mainly middle and high frequencies are in many cases preferentially encoded by neurons with higher best frequencies (Figures 6, 7A). The vocalization “tooth chatter” contains important spectral energy across the entire range of best frequencies studied and is encoded uniformly well across best frequencies. For some vocalizations and frequencies, the match of preferred encoding and spectral content is perfect (“scream long,” Figure 7A). However, deviations exist for several frequencies and vocalizations.

Discrimination performances were averaged for all multi-unit clusters within a best frequency (1/3 octave) interval. Thus, preferences of individual multi-unit clusters might be averaged out. The found deviations could be due to different amplitude and frequency modulation preferences (Schreiner and Langner, 1988), or due to low BF neurons having higher temporal but poorer spectral resolution (vice versa for high BF neurons) (Rodriguez et al., 2010). Nonlinear processing mechanisms in the ICC (McAlpine, 2004; Escabí et al., 2005; Calabrese et al., 2011) are likely to contribute to the deviations from a linear spectral mapping. Deviations could also be explained by spatial heterogeneity of receptive fields in the ICC (Portfors et al., 2011) or by further nonlinear processing properties, such as a selectivity for specific vocalizations proposed by Portfors et al. (2009). The selectivity might be shaped by inhibition (Klug et al., 2002; Xie et al., 2005).

In future studies, the deviations which point to nonlinear processing need to be analyzed in more detail. In our analysis, the multi-unit responses were successfully used to discriminate between vocalizations. To further address our questions, it would be interesting to investigate single neuron behavior within the multi-unit clusters. Single-neuron resolution within the multi-unit cluster would allow one to answer the question whether a multi-unit cluster responds differently to different vocalizations because it either recruits different groups of neurons for each vocalization, or because the spike times of the multi-unit, and thus of the single neurons, are different for each vocalization. Single neuron recordings for which the best frequency (not a compound BF, as is the case for multi-unit clusters) and best amplitude modulation frequency are known, would allow one to compare optimal encoding to the spectral and temporal content of the vocalizations. Finally, single neuron responses would allow one to test for a possible call-selectivity to individual vocalizations (Portfors et al., 2009).

Future studies could also address neural discrimination for different exemplars of the same vocalization. Then one could test the hypothesis that neurons with a best frequency matching the vocalization's main spectral content are acoustically more discriminative than other neurons. An alternative hypothesis would be that the spectral changes between the different trials are important enough and that each trial is optimally discriminated according to its individual main spectral content. This might give insights into how the variability of the vocalization's variants manifests in the neural representation, thus how small differences are represented.

We found that trends of preferred encoding for individual vocalizations and spectral content are similar in several cases. Portfors et al. (2011) have shown spatial heterogeneity of receptive fields in the mouse inferior colliculus. However, this does not preclude that vocalizations are encoded spatially along the BF-gradient. Spatial preference for encoding certain vocalizations, though linked to their spectrotemporal properties in the ICC, is reminiscent of vocalization encoding in spatially segregated columns in the auditory cortex of guinea pigs (Grimsley et al., 2012). Our results are consistent with earlier work showing that neurons in the ICC encode spectrotemporal acoustic patterns of vocalizations (Suta et al., 2003). Neural tuning to spectrotemporal modulations is optimized to efficiently encode vocalizations (Woolley et al., 2005). Furthermore, our work supports an efficient encoding strategy, suggesting that higher level neural representations match the statistical and behavioral qualities of the stimuli.

### 4.2. A small number of units encode vocalizations

We found that, in general, responses from one multi-unit cluster do not contain enough information to faithfully encode vocalizations. Combining responses of up to 3–6 multi-unit clusters significantly increases discrimination between the 11 vocalizations used, and this is supported by previous findings (Engineer et al., 2008). These neural groups comprise about 9–30 single neurons and additional background noise. Improvement of discrimination between songs has also been demonstrated when combining single neuron responses of the IC-analog in zebra-finches (Schneider and Woolley, 2010). Improvement was highest when combining single neurons with similar frequency tuning and this has been suggested to be due to reduction of trial-to-trial variability. However, we found that it did not alter discrimination significantly when multi-unit clusters with similar frequency tuning (or possibly amplitude modulation tuning) or with very different tuning were combined. Improvement of neural discrimination due to reduction of trial-to-trial variability might be less predominant for multi-unit clusters than for single neurons. This could be due to an already reduced response variability of some multi-unit clusters, since their responses are averages of adjacent single neurons and adjacent neurons have, in general, a higher probability of displaying similar spectrotemporal preferences than if they were dispersed (Chen et al., 2012b). Different tuning will add independent information to the discrimination, but adjacent units will often have similar tuning, and it seems puzzling that the small existing differences in the units' responses are important enough to contribute new information to the discrimination. This result, however, is supported by the work of Holmstrom and colleagues who found that neural responses in the mammalian ICC are heterogeneous, and that this heterogeneity appears to be enough for an efficient encoding of vocalizations (Holmstrom et al., 2010).

Combining only temporal information of the spiking responses yields higher discrimination for multi-unit clusters with similar frequency tuning than along the best frequency gradient. This indicates that mainly temporal information is encoded within sets of similarly frequency-tuned neurons, as suggested by earlier work (Schreiner and Langner, 1988).

Combined spectral and temporal information of responses from 3 to 6 multi-unit clusters along the best frequency gradient allowed, on average, for near perfect discrimination in all 72 studied recordings, and was even perfect for some of them. Thus, a few multi-unit clusters are able to reliably discriminate a representative set of behaviorally relevant vocalizations. While future studies need to be done, our findings might suggest an estimate for the order of magnitude at the level of multi-unit clusters when electrically stimulating neuronal tissue, as groups of neurons, and not single neurons, are stimulated. A future study could also investigate whether combining further multi-unit clusters might allow one to discriminate between finer differences in the vocalizations, e.g., discriminate between the same vocalization from different guinea pigs.

### 4.3. Temporal response correlations in the ICC do not facilitate encoding

Correlations of simultaneously recorded neurons have been found to be beneficial to encoding (Da Silveira and Berry II, 2013), but were also found to be destructive to encoding (Gawne and Richmond, 1993). We showed that in the mammalian ICC, temporal correlations of simultaneously responding multi-unit clusters do not contribute to better neural discrimination between vocalizations. Response correlations are stronger for simultaneous than for non-simultaneous responses and show large variability across multi-unit clusters. These findings are independent of the stimulus frequency, and hence, the response strength. That temporal correlations do not facilitate encoding is further supported by our finding that discrimination improved regardless of whether neighboring or distant neurons were combined, with neighboring neurons having a higher probability of interacting and displaying temporal correlations.

Hence temporal correlations, which might be due to interactions between the multi-unit clusters, are not beneficial to encoding. The contribution from each multi-unit cluster is diverse enough to add new information. At the level of multi-unit clusters, the output of the ICC seems to be shaped more by the input and the arrangement of receptive fields than by interactions between neural groups. Single neuron recordings might allow us to quantify the amount of correlations due to neuronal interactions, and possibly to infer structural connectivities between neurons –although clear restrictions exist (Melssen and Epping, 1987). It would be interesting in future analyses to investigate whether temporal correlations of single neurons within the multi-unit clusters are beneficial to encoding.

Multi-unit clusters in the ICC act as independent encoders in the sense that correlations with other neurons do not facilitate encoding, as demonstrated for single retinal ganglion cells (Nirenberg et al., 2001), and redundancy is minimized as suggested for central systems (Barlow, 1972).

In summary, our data show that the encoding of individual vocalizations remains good over broad frequency regions, which should be beneficial in a behavioral context. Our finding that response redundancy is minimal for small groups of encoding neurons supports an efficient encoding strategy for natural behaviorally relevant vocalizations in the mammalian inferior colliculus.

## Author note

This paper is based on a chapter in the PhD thesis of Dominika Lyzwa which had been published under the Common License agreement. https://ediss.uni-goettingen.de/handle/11858/00-1735-0000-0022-6026-D.

## Author contributions

DL and JH conceived the work. DL performed data analysis and wrote the manuscript. DL and FW interpreted the data. DL, FW, and JH revised it critically for important intellectual content.

## Funding

This work was supported by Grant # 01GQ0810 and # 01GQ0811 of the Federal Ministry of Education and Research within the Bernstein Focus of Neural Technology Göttingen.

### Conflict of interest statement

The authors declare that the research was conducted in the absence of any commercial or financial relationships that could be construed as a potential conflict of interest.
